# Author response to: Recurring bacterial strains, subclusters, and the importance of practising lessons learned

**DOI:** 10.1017/S0950268824001171

**Published:** 2024-12-16

**Authors:** G. Sean Stapleton, Joshua M. Brandenburg, Megin Nichols

**Affiliations:** Division of Foodborne, Waterborne and Environmental Diseases, Centers for Disease Control and Prevention, Atlanta, USA

**Keywords:** Salmonella, outbreak, public health, zoonotic, foodborne, surveillance

We respectfully acknowledge the letter written by Hedberg et al. [1] commenting on the processes of multistate enteric illness outbreak investigations like those reported in our publication [2]. We agree with the central thesis of Hedberg et al. that ‘we must continue to critically review our methods [of outbreak investigation] in order to improve our practice.’ The U.S. Centers for Disease Control and Prevention (CDC) continually works with our partners to critically evaluate standard processes used to coordinate multistate outbreak investigations to make improvements for the protection of public health. Following the conclusion of multistate enteric illness outbreak investigations, CDC epidemiologists formally document all findings and share these with our investigative partners across other government agencies. We annually publish public summaries of multistate investigations [3], highlight notable or novel findings in peer-reviewed journals, and present at conferences and meetings. By engaging with academic, industry, and government partners through these different forums, we routinely identify opportunities to refine our investigation strategies and implement these improvements during subsequent investigations.

Additionally, CDC regularly identifies and implements new technologies and evidence-based communication strategies for sharing data with partners and the public throughout the course of outbreaks. This has included identification of and public communication about reoccurring, emerging, or persisting strains of enteric pathogens, which require unique approaches to surveillance, outbreak response, and mitigation [4]. Also, the use of whole genome sequencing (WGS) to detect and characterize enteric pathogens is continually refined to better understand strains at a molecular level, and CDC conducts research with collaborators to understand the utility of other WGS analysis approaches *–* such as pangenome analysis *–* for public health response and prevention efforts.

We emphasize the challenges faced and improvements made in multistate response in our publication [2], which documents a strain of *Salmonella* Hadar that emerged shortly after the onset of the SARS-CoV-2 pandemic [5] and has continued to cause illnesses and outbreaks to date [6]. During that year, sales of backyard poultry increased among the public following quarantine orders [7]. After confirming that this strain caused a large outbreak that was linked to contact with backyard poultry in 2020, public health agencies subsequently confirmed genetically related *Salmonella* Hadar illnesses linked to consumption of ground turkey in 2021 [2]. As we acknowledge in our publication, findings collected during the 2021 ground turkey-associated *Salmonella* Hadar outbreak imply that some patients identified during the 2020 backyard poultry-associated *Salmonella* Hadar outbreak might have been exposed to the outbreak pathogen by contaminated food products. This could mean that additional actions might have been taken to prevent illnesses when this strain of *Salmonella* Hadar emerged. Following these outbreaks, additional multistate outbreaks of *Salmonella* Hadar have been identified, and epidemiologic data collection includes routinely interviewing patients about food poultry and backyard poultry exposures. CDC continues to actively monitor the persistence of this strain, modulate outbreak investigation approaches, collaborate with partners to research this strain, and solicit feedback [6].

In summary, we agree with Hedberg et al. [1] regarding the importance of evaluating outbreak investigation methods and appreciate the opportunity to highlight the multiple steps and continuous improvement initiatives that CDC and partners take to review multistate outbreak investigations and implement changes as necessary for the benefit of public health and food safety.
